# Immune Checkpoint Inhibitor-Related Myositis: From Biology to Bedside

**DOI:** 10.3390/ijms21093054

**Published:** 2020-04-26

**Authors:** Antonio G. Solimando, Lucilla Crudele, Patrizia Leone, Antonella Argentiero, Matteo Guarascio, Nicola Silvestris, Angelo Vacca, Vito Racanelli

**Affiliations:** 1 IRCCS Istituto Tumori “Giovanni Paolo II”, 70124 Bari, Italy; antonio.solimando@uniba.it (A.G.S.); argentieroantonella@gmail.com (A.A.); n.silvestris@oncologico.bari.it (N.S.); 2 Department of Biomedical Sciences and Human Oncology, Unit of Internal Medicine “Guido Baccelli”, University of Bari Medical School, 70124 Bari, Italy; lucillacrudele@gmail.com (L.C.); patrizia.leone@uniba.it (P.L.); guarasciomatteo@gmail.com (M.G.); angelo.vacca@uniba.it (A.V.)

**Keywords:** immune checkpoint inhibitors, immune-related adverse events, myositis, dermatomyositis, polymyositis, autoimmunity, cancer

## Abstract

Immune checkpoint inhibitor (ICI)-related inflammatory diseases, including polymyositis (PM) and dermatomyositis (DM), in patients suffering from neoplastic disorders represent a medical challenge. The treatment of these conditions has taken on new urgency due to the successful and broad development of cancer-directed immunological-based therapeutic strategies. While primary and secondary PM/DM phenotypes have been pathophysiologically characterized, a rational, stepwise approach to the treatment of patients with ICI-related disease is lacking. In the absence of high-quality evidence to guide clinical judgment, the available data must be critically assessed. In this literature review, we examine partially neglected immunological and clinical findings to obtain insights into the biological profiles of ICI-related PM/DM and potential treatment options. We show that differential diagnosis is essential to stratifying patients according to prognosis and therapeutic impact. Finally, we provide a comprehensive assessment of druggable targets and suggest a stepwise patient-oriented approach for the treatment of ICI-related PM/DM.

## 1. Introduction

Immune surveillance has emerged as a pivotal issue in the invasiveness of both visceral [1] and skeletal [2,3] malignancies, as it fuels a vicious cycle between the neoplastic cells and the immune microenvironment. Immune checkpoint inhibitor (ICI)-based approaches, aimed at interrupting corrupted immune bystander cells and reactivating an effective anti-cancer response, represent one of the most significant therapeutic innovations in the oncologic landscape to date [4] with an ability to target solid [5,6] and hematological [7,8] malignancies. However, among the side effects of ICI therapy is ICI-related polymyositis (PM), an inflammatory process affecting the skeletal muscles. While this condition is rare, it can be severe and potentially deadly, as it may cause rhabdomyolysis in striated muscle, including the myocardium. PM can occur as a reactivation of a previous paraneoplastic polymyositis or dermatomyositis (DM) or as a new entity [9,10]. Clinically, PM/DM manifests as worsening muscle weakness and myalgias. Compared to non-ICI-related forms of inflammatory myositis, oculomotor and axial muscle involvement, including diplopia and muscular weakness as suggestive symptoms, have been reported [11,12,13,14]. An involvement of the bulbar musculature can cause dysarthria, dysphonia or dysphagia. Physical examination will reveal skin signs suggestive of DM, while a careful history can rule out alternative causes, such as chronic steroid myopathy. Blood chemistry tests support the diagnosis by detecting elevated serum levels of muscle damage markers (creatine phosphokinase, lactate dehydrogenase, transaminases, aldolases) and in some patients myositis-specific or myositis-related antibodies. ICI-therapy-related variants are characterized by the frequent involvement of other targets of the peripheral nervous system and of the myocardium (myasthenia gravis [MG], polyradiculoneuritis, myocarditis), which can be detected using specific tests: increased troponin levels suggest cardiac involvement; an electromyoneurography (EMG/ENG) study can confirm the presence of myogenic damage or the presence of neuropathic damage or neuromuscular plaque disease. Further information might be obtained with magnetic resonance imaging (MRI) of the muscle and/or muscle biopsy; the latter can identify secondary manifestations, such as giant cell arteritis, systemic lupus, and sarcoidosis.

In a meta-analysis of adverse events related to ICI, the incidence of grade 3–5 adverse events involving the central nervous system (encephalitis, encephalopathy, aseptic meningitis or myelitis) was 0.46% (22 of 4775 ICI-treated patients in 12 studies). In the same analysis, the incidence of peripheral neuropathy of any degree was 5% (220 of 4390 patients exposed to ICI in 17 studies), significantly lower than that occurring in association with conventional chemotherapy. Among 3128 patients from eight studies, the meta-analysis found four cases of grade 3–5 MG (0.13%) and three of grade 3–5 myositis (0.10%) [15]. However, the clinical scenario is often multifaced, being associated with several other uncommon features. Indeed, the presence of bulbar symptoms, dysphagia, or ocular motor symptoms can suggest the diagnosis of MG that it is not confirmed by antibody levels and/or nerve stimulation tests. A small portion have also cardiac myositis, which turns out to be among the most fatal of all the irAEs. Any of these three compartments of myasthenia-skeletal muscle myositis or carditis can occur individually or all together, variably impacting the patient outcome.

### 1.1. Biological Background: Bridging the Gaps between Immune Checkpoint Inhibition and Physiopathology ICI-Related Disease

Among the functions of the human immune system is tumor surveillance. In this stepwise response, antigen-presenting cells (APCs) control the tumoral antigen load by priming and activating T cells, which in turn recognize and then destroy the malignancy, thus releasing an even higher tumoral antigen load, which again elicits an immune response. However, when a single step in this process is impaired, the tumor can grow exponentially. Immune-directed therapy attempts to restore the homeostatic equilibrium [16]. One of the pharmacodynamically most successful ICI-related approaches to enhancing the anti-tumor immune response consists of interfering with the negative costimulation of T cells, by inhibiting PD-1, PD-L1, and CTLA-4 [17]. PD-1 (CD279) is a type I transmembrane receptor expressed on the surfaces of T cells, B cells, monocytes, natural killer cells, and dendritic cells. It has two physiological ligands: PD-L1 (B7-H1, CD274) and PD-L2 (B7-DC, CD273). These Ig-like transmembrane receptors are also expressed on the cell surface [17]. CTLA-4 is a CD28 homolog with a high affinity for B7-1/2. The binding of CTLA-4 to B7-1/2 acts as a co-inhibitory signal that hinders early T cell activation [18]. A study in CTLA-4-deficient animals demonstrated the pivotal role of CTLA-4 in halting T-cell-mediated immune anti-tumor activity [19]. Inborn immunity errors of immune checkpoints similarly predispose patients to autoimmune manifestations. For example, in CHAI/LATAIE, a type of CTLA-4 insufficiency, the clinical features mirror those of ipilimumab-related responses [19]. ICI modulators interrupt T cells and APCs, but also cancer cells. The immune checkpoints involved in these processes are also relevant in the pathogenesis and treatment of rheumatic diseases. The difference is that while in cancer therapy the negative stimulation is halted by ICI administration, in the treatment of autoimmunity, negative costimulation is promoted [9].

**Figure 1:**
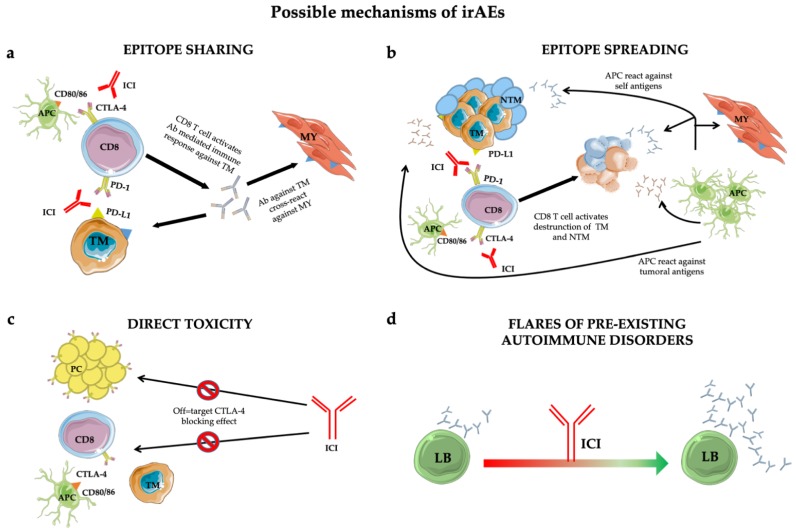
Mechanisms of immune-related adverse events (irAEs): epitope-sharing (**a**); -spreading (**b**); direct toxicity (**c**) and flares of pre-existing autoimmune disorders (**d**). See text for details. Abbreviations: ICI = immune checkpoint inhibitor; TM = tumoral cell; MY = myocytes; Ab = antibody; NTM = non tumoral cell; APC = antigen presenting cell; PC = pituitary cell; LB = B lymphocyte.

However, in the anti-tumoral activity of ICIs, a normal inflammatory reaction is elicited that can lead to immune-related adverse events (irAEs). The biological mechanisms by which those adverse events take place are still poorly understood [13] but several pathophysiological pathways have been proposed (Figure 1).

Treg cells are responsible for self-tolerance and constitutively express CTLA-4 on their surfaces [20]. By triggering antibody-dependent cellular cytotoxicity (ADCC)-mediated Treg depletion, CTLA-4 antagonists increase the T effector (Teff) cell proliferation and thus an immune response to the tumor [21]. PD-L1 antibodies break the link between PD-L1 and Treg, thus also reducing Treg generation. Depending on the corrupted equilibrium between Treg and Teff cells, a loss of peripheral tolerance induced by ICIs can drive not only the desired anti-tumor immune response but also autoimmune adverse events [21].

Additionally, the epitope-sharing (Figure 1a) between tumor and healthy tissue was shown to be the cause of a cross-reaction inducing Teff activation against self-tissues, evidenced in a patient who developed a lymphocyte infiltrate in heart and skeletal muscle upon ICI treatment [22].

An alternative mechanism leading to irAEs is represented by the epitope spreading (Figure 1b) [23], in which Teff-cell-mediated tumor cell death in the cancer-microenvironment causes the release of a huge amount of tumor antigens and self antigens. The resulting autoimmune reaction induces an uncontrolled propagation of this response [18]. 

Furthermore, autoptic evidence suggests that direct toxicity (Figure 1c) [24] can also trigger irAEs. This was shown in a subset of adenohypophyseal endocrine cells whose CTLA-4 expression mirrorred that of T cells. Consequently, therapeutic administration of an anti-CTLA-4 monoclonal antibody with antitumor effects could elicit type II and type IV hypersensitivity and hypophysitis. 

Finally, preclinical predisposition to autoimmunity constitutes an exacerbation-initiating factor per se (Figure 1d) [25]. Patients with rheumatoid factor and auto-antibody positivity without clinical manifestations before treatment are more likely to develop irAEs. However, insights into the true impact of autoimmune substrates are lacking due to the exclusion from ICI trials of patients with autoimmune diseases. However, retrospective case series found no significant difference in the incidence of flares in patients with and without pre-existing autoimmune disorders [26,27,28,29,30].

### 1.2. Characteristic Autoantibody Patterns

The antibodies characteristic of inflammatory myopathies [31] are conventionally divided into those that are myositis-associated (MAAs) vs. myositis-specific (MSAs) [32]. Antibodies in the first group are a common feature of overlap syndromes involving other systemic autoimmune diseases [33], particularly scleroderma and systemic sclerosis. The best characterized antibodies are those that recognize the 52-kDa Ro/SSA antigen, a ribonucleoprotein associated with a complex that binds small RNA molecules; the DNAPK antigen (Ku/DNA-dependent protein kinase), a kinase necessary for DNA repair; and PM-Scl, a complex of 11 proteins with nucleolar localization that comprises the autoantigen of PM/scleroderma. The second group includes antibodies directed against histidyl transfer-RNA-synthetase (Jo-1) [34], the most frequently occurring antibodies. The latter are among a group of eight autoantibodies directed against aminoacyl-transfer-RNA-synthetase, which catalyses the binding of specific amino acids to transfer RNA. The presence of these autoantibodies identifies a subset of patients suffering from “anti-synthetase syndrome”. Autoantibodies that bind the SRP (signal recognition particle) are also members of the second group. SRP is an RNA-protein cytoplasmic complex that recognizes secreted and membrane-associated proteins in addition to regulating the translocation of proteins through the endoplasmic reticulum. Anti-SRP autoantibodies are associated with particularly severe myositis, extensive necrosis, and an unfavorable prognosis. Anti-Mi-2 antibodies recognize a helicase that is part of the *NuRD* (nucleosome remodeling deacetylase) complex, which plays a key role in gene transcription. While anti-Mi-2 antibodies are very specific for DM and generally associated with a favorable prognosis, they also increase the risk of cancer [35].

DM is associated with several antibodies targeting melanoma differentiation antigen 5 (*MDA5*) [36] and transcriptional intermediary factor 1 (*TIF1*) [37]. Patients positive for anti-MDA5 antibodies often have high-grade palmar rash, digital ulcers, rapidly progressive interstitial lung involvement, and amyopathic DM [38] whereas those with anti-TIF1 and anti-NXP2 antibodies have an increased risk of malignancies [39].

In most of cases with ICI-related myositis, MSAs or MAAs were undetected. Nevertheless, in a subgroup of ICI-treated patients, enrolled in clinical trials, banked serum demonstrated pretreatment auto-antibody positivity. Since the presence of asymptomatic autoantibody, upon ICI treatment, can be followed by an explosive disease, an asymptomatic phenotype might constitute the early irAEs phase in subjects genetically predisposed to full-blown disease [40,41]. In the frame of this thinking, ICI candidate baseline screening might contribute to detect autoimmune predisposition. To this end, real life studies might be worthy, since most patients with autoimmune disease were censored from clinical trials.

## 2. Clinical Work-Up

Patients on ICI therapy must undergo neurological and muscular evaluation in the case of new-onset symptoms or a worsening of pre-existing neuro-muscular symptoms (Table 1). However, the diagnostic process is often complicated by pre-existing neuro-muscular involvement as well as by the presence of systemic conditions and metastases.

Thus, the first objective must be the exclusion of other possible causes (progression of oncological disease, infectious, metabolic, autoimmune, paraneoplastic, or other neuro-muscular syndromes). Since there are no biological and clinical markers pathognomonic for ICI-related myositis, the differential diagnosis remains the most informative component in the stepwise approach to a patient in whom myositis is strongly suspected on clinical grounds [43]. The temporal relationship of the symptoms to ICI treatment and the presence of radiological and/or bio-humoral signs of inflammation are useful in ruling out other causes (Table 2). Particular emphasis should guide the differential diagnosis approaching the patient with paraneoplastic manifestation due to muscular involvement. While all systemic conditions can be relatively easily differentiated from irAEs based on laboratory tests, the differential diagnosis between ICI-related and paraneoplastic muscular involvement can be challenging. The clinical signs of cancer progression and antibody positivity can support the approach to the patient with muscular involvement and cancer under immunotherapeutic treatments, by guiding the differential diagnosis between paraneoplastic manifestation and ICI-related myopathies. Both syndromes are associated with malignancies, nonetheless the paraneoplastic syndrome characteristically correlates with tumour progression and specific immune-laboratories pathways, detectable by performing MAAs and MSAs tests [44]. 

A clinically relevant differential diagnosis should include paraneoplastic PM and DM, which constitute inflammatory myopathies characterized by weakness of the proximal limb muscles and pain of variable degree [45]. In general, in PM/DM, there is a progressive weakening of the muscles, especially at the level of the hips and thighs. The purple skin rash (heliotrope) seen in some patients is typically localized to the eyelids, bridge of the nose, cheeks, forehead, chest, elbows, knees, and around the nail bed [46]. Laboratory tests characteristically show an increase in creatine phosphokinase (CPK). The electromyographic pattern will suggest a myopathic process and inflammatory infiltrates, whereas muscle biopsy will reveal necrosis and atrophy of the fibers. Those conditions are found in association with cancer in 10% of patients [45,46]. The malignancy can precede or follow the onset of myositis for about two years. The incidence of cancer is higher in PM/DM patients over the age of 60, such that in this population all tests to localize and identify the neoplasm are necessary. The highest prevalence (globally about 90% of the reported cases) of ICI-induced myositis is observed in lung (NSCLC), melanoma, and genitourinary tumors [11,12,41,47]. Conversely, paraneoplastic PM/DM are most frequently present in breast and lung tumors [46]. Remarkably, the proportion of major cancers related to ICI-related myositis might be biased by the prevalence of ICI treatment in a selected oncologic population. The broader diffusion of ICI treatment might impact the irAE epidemiologic landscape in the future.

### 2.1. Treatment of ICI-Induced Myositis: Corticosteroids

A multicenter case series published by Moreira et al. reported 20 cases of myositis (including those overlapping with MG, polyneuropathy, and myocarditis). Fifteen patients (79%) were treated with corticosteroids (unspecified), with a complete resolution of symptoms achieved in 50% [47]. In the series reported by Touat et al. [11], nine of 10 patients with ICI-related myositis underwent corticosteroid treatment (five with methylprednisolone 0.5–1 g/day i.v., four with prednisone 1 mg/kg/day orally), including three patients who received corticosteroids in combination with intravenous immunoglobulin (IVIG) or plasmapheresis. All patients experienced clinical improvement, as determined by reductions in the Rankin score (mRS; medium mRS pretreatment: 3, medium mRS post-treatment 0.67) and biomarker levels as well as by a normalization of CPK values, within a median of 44 days. A case series published by Seki et al. in 2019 [12] described 19 patients with anti-PD-1 myositis (grade 2 in 10 patients, grade 3–5 in nine patients). Of the 17 patients (89%) treated with corticosteroids, nine (47%) received pulse methylprednisolone intravenously and oral tapering with prednisolone, and eight (42%) were treated with oral prednisolone (maximum average dose 38.75 mg/day). The five patients with severe PM (ECOG performance status ≥3) received an additional line of therapy (IVIG in four patients, plasmapheresis in four, tacrolimus in one patient). After an average follow-up of 12 months, remission was reported in 53% (*n* = 10) and clinical improvement in 42% (*n* = 8). In a non-systematic review published in 2019, Kadota et al. [41] surveyed public datasets and identified 15 reports of ICI-related myositis, treatment, and clinical outcome. Five patients had concomitant myocarditis, and two had concomitant acetylcholine-receptor-positive (AChR) MG. All patients were treated with corticosteroid (posology not reported), in combination with IVIG in 40% (*n* = 6), plasmapheresis/plasma exchanges in 40% (*n* = 6), and infliximab in 13% (*n* = 2). Improvement was reported in 10 patients (67%). In the 2019 systematic review by Johansen et al. [48], 29 patients were considered to have ICI-related myopathy; 55% were treated with corticosteroid i.v. and 31% with oral corticosteroid. Despite the available evidence, the data are limited such that high-quality indications and guidelines for the treatment of myositis remain an unmet medical need.

However, the current therapeutic regimen in most patients consists of corticosteroids in those with grade 1–2 ICI myositis, such as oral prednisone 0.5–1 mg/kg/day followed by oral tapering. In patients with grade 3–4 ICI-related myositis, treatment with i.v. methylprednisolone 1 g/day for five days, followed by oral tapering (starting from prednisone 1.5 mg/kg/day) should be considered.

### 2.2. Treatment of ICI-Induced Myositis: Immunoglobulins and Plasmapheresis

Specific studies on the efficacy of IVIG or plasmapheresis treatment in patients with ICI-related myositis have yet to be conducted. Touat et al. and Moreira et al., in two independent case series, showed that IVIG was beneficial in patients with ICI myositis when provided in association with corticosteroids [11,47]. Seki et al. [12] reported the clinical benefit of plasmapheresis, either alone or in combination with IVIG. In their literature review, Kadota et al. [41] found that IVIG and plasmapheresis were effective when used in combination with other drugs, such as infliximab plus plasmapheresis. The systematic review by Johansen et al. [48], which included the cases reported by Kadota et al. [41], provided a comprehensive assessment of the immunological approaches to neuromuscular ICI-related side effects, including the effectiveness of IVIG and plasmapheresis. However, analyses of the potential benefit of corticosteroid treatment are complicated by the difficulty in extrapolating statistically powered indications. Thus, clinicians should be guided by efficacy data based on the available reports as well as their own clinical judgment.

In patients with steroid-refractory non-ICI-related inflammatory myopathies, IVIG has demonstrated clinical efficacy in terms of muscle strength [49]. Subcutaneous administration is a feasible alternative [50] and can be considered in some patients, especially those with coexisting primary [51,52] or secondary immunoparesis [53,54]. 

The use of plasmapheresis in combination with cyclophosphamide and chlorambucil to treat non-ICI-related forms of inflammatory myopathy showed promising results in a historical study of 35 patients not responsive to previous treatments (improvement of muscle strength in 32/35) [55]. However, this benefit was not confirmed by a subsequent randomized controlled trial of 39 patients [56]. In some patients, infliximab and extracorporeal immunoadsorption may be valuable options. Sporadic reports suggested alternative options for patients with glucocorticoid-refractory disease and/or during tapering, including the use of methotrexate, mycophenolate mofetil, azathioprine, and hydroxychloroquine, frequently in combination with IVIG and plasma exchange [57].

**Figure 2:**
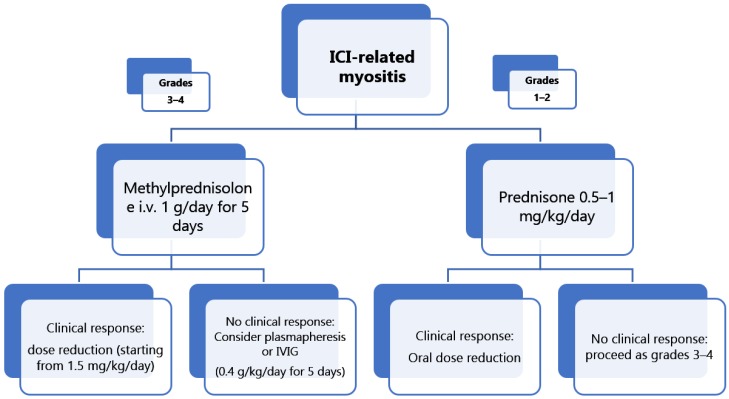
Integrated approach to ICI-related myositis patients according to clinical grade.

Unlike corticosteroid therapy, not all hospitals are able to offer plasmapheresis and IVIG. Nonetheless, both should always be considered when irAEs are severe and the clinical response to glucocorticoid is unsatisfactory (Figure 2).

In 5% of patients with PM/DM and concomitant ocular symptoms of MG, symptomatic effectiveness, in terms of both extraocular and oculobulbar motility, was demonstrated with pyridostigmine [57].

Refractoriness to the symptomatic approach and continued clinical severity can motivate a temporary discontinuation of ICI treatment, particularly in patients with the 3M (myocarditis, myositis, and MG) syndrome), whose clinical course is aggressive and characterized by a poor prognosis [57]. This clinical scenario requires a comprehensive stepwise assessment that takes into account prognostically relevant organ damage and must include a trans-thoracic echocardiogram (ECHO) [58]. Indeed, the clinical relevance of myocardial involvement, evaluated by ECHO, has been demonstrated, especially during early-stage evaluations, and may point to immune-related mechanisms [11,59]. In patients with aggressive, non-responsive disease, particularly when associated with myocardial involvement, off-label treatment should be considered, such as abatacept [60], at a dose of 500–1000 mg every two weeks [61], and B-cell depletion [62]. However, as quality evidence supporting these approaches is scant, the therapeutic backbone consists of rituximab-based therapies [63,64], currently 375 mg/m^2^ per week for four weeks [65,66]. Recently, expert panels evaluated the benefits of rituximab in patients with disease refractory to glucocorticoid and to at least to one of the following: methotrexate, azathioprine, mycophenolate mofetil, and intravenous immunoglobulin. The assessed dose of rituximab was 750 mg/m^2^ (up to 1 g) i.v. followed by a second infusion two weeks later, and repeat courses (375 mg/m^2^ as a single administration or with a second infusion two weeks apart) every 6–18 months as required [67]. The most comprehensive data regarding treatment of ICI-induced myositis and the related clinical outcome are summarized in Table 3.

## 3. Clinical Outcome and ICI-Related Muscular Involvement

A high morbidity and mortality of patients with MG and ICI-related myositis was reported by Anquetil et al. [62] based on a VigiBase analysis. The authors identified 180 reports of myositis in the course of ICI. In those patients, mortality was significantly higher than in patients with idiopathic autoimmune myopathies (21.2 vs. < 10%). Severe complications (defined as prolonged hospitalization, life-threatening event or residual disability) occurred in 49.4%. Johansen et al. similarly found a high mortality in patients with ICI-related myositis (12 out of 29 patients, 41%) [48]. Most likely, the higher mortality rate can be explained by the existence of a multifaced clinical scenario, being associated with several other uncommon features for spontaneous autoimmune myopathies. Moreover, additional irAEs often parallel ICI-related myositis, by an immune-mediated storm including myocarditis, hepatitis, colitis, lung involvement, and nephritis [40]. Nevertheless, clinical outcome appears to be driven primarily by the cardiac involvement and by the cancer-related events [11,41,47,48,58]. However, the available data seem to be affected by the lack of homogeneity of the follow-up length of time; often it is difficult to dissect the ICI-induced myositis prognostic impact from the ancillary irAEs (Table 3). High-quality data able to estimate the specific impact of mortality exerted by ICI-related myositis are lacking. Nonetheless, the low incidence of muscular adverse events compared to irAEs will represent a clinical challenge.

Remarkably, the occurrence of an irAE and of ICI-related myositis in some retrospective studies deemed significantly correlated with deeper tumor response: enhanced efficacy while controlling immune-related muscle toxicity appears crucial, since irAEs may impact the clinical outcome by mirroring increased anti-cancer activity [68,69]. Collectively, standardized prospective statistically power will allow an accurate estimation of clinical outcome and treatment response. 

## 4. Conclusions

A loss of immune tolerance is the major driver of ICI-induced myositis. While the pathophysiology of ICI-induced myositis has yet to be fully elucidated, the mechanism seems to be distinct from that leading to autoimmunity, despite similarities between the two conditions. Among the mechanisms of ICI-induced myositis implicated thus far are Treg dysregulation, epitope/sharing/spreading, direct toxicity, and pre-existing auto-immunity. Biomarker identification and personalized treatment aimed at minimizing toxicity while maintaining therapeutic efficacy remain unmet medical needs and thus merit further research and clinical efforts. 

## Figures and Tables

**Table 1:** Classification of the severity of ICI-related myositis [42].

G1	G2	G3	G4
Mild pain	Moderate pain, associated with weakness; pain, that limits age-appropriate activities of daily life	Pain associated with severe weakness, that limits age-appropriate activities of daily life	Life-threatening implications

**Table 2:** Diagnostic approach to ICI-related PM/DM.

**Any Grade**
Neurological and rheumatological anamnesis, rheumatological (inspection of the skin to identify signs suggestive of dermatomyositis) and neurological (muscle strength determination) objective examinations
Blood chemistry tests including: CPK, transaminases (AST, ALT), LDH, aldolase Cardiac enzymes (to identify possible concomitant myocarditis) Markers of inflammation (ESR, CRP) Consider searching for anti-AChR antibodies (to identify possible concomitant MG) and for antibodies causing neurological syndromes and paraneoplastic myositis
Consider a neurophysiological examination (needle EMG, neuromuscular plaque determination to identify possible concomitant MG, or a nerve conduction study to identify possible concomitant neuropathy)
Consider muscle MRI or tissue biopsy if the diagnosis is uncertain
CPK, ESR and CRP for follow-up
**Grade 2**
In addition to the above: Rapid rheumatological or neurological evaluation
**Grade 3**
In addition to the above: Urgent rheumatological or neurological evaluation

ICI, immune checkpoint inhibitor; PM/DM, polymyositis/dermatomyositis; CPK, creatine phosphokinase; AST, aspartate aminotransferase; ALT, alanine aminotransferase; LDH, Lactate dehydrogenase; ESR, erythrocyte sedimentation rate; CRP, C-reactive protein; AChR, acetylcholine receptor; MG, myasthenia gravis; MRI, magnetic resonance imaging.

**Table 3:** Comparison of clinical features, treatment and outcome for ICI-related myositis.

Reference	Type of irAE n. pts	irAE Grade CTCAE	Autoantibody Subtypes n. pts Positive/Tested	Treatment n. pts	Outcome of irAE n.pts (%)
Touat et al.; 2018 [11] *n* = 10 pts	PM = 10 DM = 0	G≥3 = 9 G≤2 = 1	MSA *n* = 0/7 MAA *n* = 0/7 Other Abs: anti- SSA/Ro52 *n* = 1 *	None *n* = 1 Prednisone monotherapy *n* = 4 IVMP monotherapy *n* = 2 IVMP + IVIG *n* = 2 IVMP + PLEX *n* = 1	Remission *n* = 10 (100%) Sequele *n* = 0 (0%) irAE-death *n* = 0 (0%) All causes-death *n* = 5 (50%)
Moreira et al.; 2019 [47] *n* = 20 pts	PM = 19 DM = 1	G≥3 = 12 G≤2 = 8	MSA *n* = 4/18 MAA *n* = 1/18 anti-SRP *n* = 1 anti-TIF1γ *n* = 1 EJ + RO52 ^§^ *n* = 1 PL7 + PL12 + anti-SRP *n* = 1 Other Abs: ANA *n* = 1	None *n* = 4 Steroid monotherapy *n* = 10 Steroid + IVIG *n* = 4 Steroid + pyridostigmine *n* = 1	Remission *n* = 11 (55%) Sequele *n* = 4 (20%) N/A *n* = 3 (15%) irAE-death *n* = 2 (10%) All causes-death *n* = 3 (15%)
Seki et al.; 2019 [12] *n* = 19 pts	PM = 19 DM = 0	G≥3 = 9 G≤2 = 10	MSA *n* = 0/19 MAA *n* = 0/19 Other Abs: anti-SM *n* = 11 anti-SM + anti-AChR *n* = 2	None *n* = 2 PSL monotherapy *n* = 6 IVMP + PSL *n* = 6 PSL + IVIG *n* = 1 PSL + PPH *n* = 1 IVMP + PSL + PPH + IVIG *n* = 2 IVMP + PSL + PPH + IVIG + tacrolimus *n* = 1	Remission *n* = 10 (53%) Sequele *n* = 8 (42%) irAE-death *n* = 1 (5%) All causes-death *n* = 7 (37%)
Kadota et al.; 2019 [41] *n* = 15 pts	PM = 15 DM = 3	N/A	MSA *n* = 3/10 MAA *n* = 0/10 anti-SRP + anti-ARS *n* = 1 anti-TIF1-γ *n* = 1 anti-ARS *n* = 1 * Other Abs: anti-SM *n* = 1 + 1 * ANA *n* = 1 anti-AChR *n* = 1 + 1 *	PSL monotherapy *n* = 6 PSL + IVIG *n* = 2 PSL + PLEX *n* = 2 PSL + IFX *n* = 1 PSL + IVIG + PPH *n* = 1 PSL + IVIG + PPH + IFX *n* = 1 PS + IVIG + PLEX + pyridostigmine *n* = 2	Remission/ Improvement *n* = 10 (67%) irAE-death *n* = 5 (33%) All causes-death *n* = 7 (47%)
Johansen et al.; 2019 [48] *n* = 29 pts	PM = 29 DM = 0	N/A	MSA = N/A MAA = N/A Other Abs: anti-AChR *n* = 2/10	Steroid PO *n* = 9 Steroid IV *n* = 16 IVIG *n* = 7 PLEX *n* = 6 Pyridostigmine *n* = 1 Unspecified Immunomodulator *n* = 4	Remission/Improvement *n* = 20 (69%) irAE-death *n* = N/A All causes-death *n* = 12 (41%)

Abbreviations: pts, patients; n, number; Abs, Autoantibodies; irAE, immune-related adverse event; CTCAE, Common Terminology Criteria for Adverse Events; PM, Polymyositis; DM, Dermatomyositis; IVMP, Intravenous Methylprednisolone; IVIG, Intravenous Immunoglobulin; PPH, Plasmapheresis; PLEX, plasma exchanges; PSL, Prednisolone; IFX, Infliximab; N/A, not available; MSA, myositis-specific autoantibodies; MAA, myositis-associated autoantibodies; SM, striated muscle; SSA/Ro52, Anti-Sjögren’s-syndrome-related antigen A/Ro52; ANA, antinuclear antibody; AChR, acetylcholine receptor; ARS, aminoacyl-tRNA synthetase; SRP, signal recognition particle; TIF1-γ, transcription intermediary factor 1-γ. * Autoantibodies were pre-existing before initiation of ICI. ^§^ RO52 is included in MAA.
